# The multi-kingdom microbiome of the goat gastrointestinal tract

**DOI:** 10.1186/s40168-023-01651-6

**Published:** 2023-10-02

**Authors:** Yanhong Cao, Tong Feng, Yingjian Wu, Yixue Xu, Li Du, Teng Wang, Yuhong Luo, Yan Wang, Zhipeng Li, Zeyi Xuan, Shaomei Chen, Na Yao, Na L. Gao, Qian Xiao, Kongwei Huang, Xiaobo Wang, Kuiqing Cui, Saif ur Rehman, Xiangfang Tang, Dewu Liu, Hongbing Han, Ying Li, Wei-Hua Chen, Qingyou Liu

**Affiliations:** 1 Guangdong Provincial Key Laboratory of Animal Molecular Design and Precise Breeding, School of Life Science and Engineering, Foshan University, Foshan, 528225 China; 2 Guangxi Vocational University of Agriculture, Nanning, Guangxi 530007 China; 3 Department of Bioinformatics and Systems Biology, Key Laboratory of Molecular Biophysics of the Ministry of Education, Hubei Key Laboratory of Bioinformatics and Molecular-imaging, Center for Artificial Biology, College of Life Science and Technology, Huazhong University of Science and Technology, Wuhan, 430074 Hubei China; 4 State Key Laboratory for Conservation and Utilization of Subtropical Agro-Bioresources, Guangxi University, Nanning, 530005 China; 5 Hainan Key Lab of Tropical Animal Reproduction and Breeding and Epidemic Disease Research, College of Animal Science and Technology, Hainan University, Haikou, 570000 Hainan China; 6 Animal Husbandry Research Institute of Guangxi Zhuang Autonomous Region, Nanning, 530001 Guangxi China; 7 State Key Laboratory of Animal Nutrition, Institute of Animal Science, Chinese Academy of Agricultural Sciences, Beijing, 100193 China; 8 South China Agricultural University, Guangzhou, 510642 China; 9 College of Animal Science and Technology, China Agricultural University, Beijing, 100193 China; 10 Institution of Medical Artificial Intelligence, Binzhou Medical University, Yantai, 264003 China

**Keywords:** Goat, Gastrointestinal tract, Microbiome, Metagenome-assembled genomes, Bacteriome, Archaeome, Virome, Bacteriophages, Plant fiber digestion, Methane production

## Abstract

**Background:**

Goat is an important livestock worldwide, which plays an indispensable role in human life by providing meat, milk, fiber, and pelts. Despite recent significant advances in microbiome studies, a comprehensive survey on the goat microbiomes covering gastrointestinal tract (GIT) sites, developmental stages, feeding styles, and geographical factors is still unavailable. Here, we surveyed its multi-kingdom microbial communities using 497 samples from ten sites along the goat GIT.

**Results:**

We reconstructed a goat multi-kingdom microbiome catalog (GMMC) including 4004 bacterial, 71 archaeal, and 7204 viral genomes and annotated over 4,817,256 non-redundant protein-coding genes. We revealed patterns of feeding-driven microbial community dynamics along the goat GIT sites which were likely associated with gastrointestinal food digestion and absorption capabilities and disease risks, and identified an abundance of large intestine-enriched genera involved in plant fiber digestion. We quantified the effects of various factors affecting the distribution and abundance of methane-producing microbes including the GIT site, age, feeding style, and geography, and identified 68 virulent viruses targeting the methane producers via a comprehensive virus-bacterium/archaea interaction network.

**Conclusions:**

Together, our GMMC catalog provides functional insights of the goat GIT microbiota through microbiome-host interactions and paves the way to microbial interventions for better goat and eco-environmental qualities.

Video Abstract

**Supplementary Information:**

The online version contains supplementary material available at 10.1186/s40168-023-01651-6.

## Background

The goat (*Capra hircus*) is an economically important livestock animal across the world [1, 2] and plays an indispensable role in human life by providing meat, milk, fiber, and pelts [3]. Today, about 1000 goat breeds and more than one billion goats are kept globally according to the Food and Agriculture Organization (FAO) of United Nations [4]; among all countries, China ranks among the highest in the world with about 140 million goats (http://www.fao.org/corp/statistics/en/).

Microbial consortia in the herbivore gastrointestinal tract (GIT) have important functional roles for their ruminant hosts; for example, forage grass, hay, corn, and silage could be first processed by the rumen microbiota and then utilized by the hosts [5]. Previous studies had focused on lignocellulose breakdown and their relation to rumen microbiota [6, 7], but recent studies showed that GIT microbiota could have significant effects in the overall food digestion and nutrient absorption [8–10]. In our study in buffalo, we reported that the cellulose-digesting flora changed along the digestion of buffalo gastrointestinal tract [10]. It is thus conceivable that the microbial compositions are different at different GIT sites and often associate with the functions of the latter. In addition to the GIT sites, other factors are known to contribute significantly to host-associated microbiomes, including age [11], feeding style [12], geographical location [13], and host species [10, 14–17].

A useful strategy to explore novel microbial linages is metagenomic next-generation sequencing (mNGS) on environmental samples, followed by the reconstruction of metagenome-assembled genomes (MAGs). This strategy has been recently used to reconstruct MAGs from pig [18], chicken [19, 20], mouse [21], cattle [22], buffalo [10], ruminants [9], and human [23], which offered the researchers the opportunity for quickly accessing these unexplored microbiomes and revealing functional interactions between the microbial ecology and the GIT sites of interest. However, despite a few studies on individual GIT sites [7, 24, 25] or using a few animals [9], systematic exploration of the microbial ecology across all goat GIT sites with a large number of goats is still unavailable.

In this work, we present a comprehensive survey (497 samples) on the microbial ecology covering different GIT sites, ages, feeding styles, and geographical locations from 268 goats. We submitted these samples for mNGS and generated ~ 3 Tb of raw sequence data. We reconstructed a total of 4075 bacterial and archaeal genomes metagenome-assembled genomes (MAGs) and 7204 viral genomes, and annotated 4,817,256 non-redundant protein-coding genes. We found that 43.71% (*n* = 1781) of the MAGs and 90.91% (*n* = 6549) of the viral genomes were novel under the threshold of 95% average nucleotide identity (ANI) with public genomic datasets, and 20.70% (*n* = 997,417) of the proteins had no homologs in public protein databases (eggNOG and CAZyme databases), indicating novelty of our datasets. Through comparative metagenomic analysis, we identified known and novel associations between microbes and the goat GIT sites and investigated the contributions of environmental and host factors to the microbial diversity. We constructed a comprehensive virus-bacterium/archaea interaction network and identified 68 lytic viruses targeting the methane-producing species. Together, we filled the gap in goat microbial ecology research by providing catalogs of multi-kingdom microbial (bacterial, archaeal, and viral) genomes and encoded-proteins. We believe these results and resources will facilitate further studies on functional and/or phenotypical impacts of microbiota in goat and related ruminants, and pave the way to microbial interventions for better goat production and eco-environmental quality.

## Methods

### ***Sample collection***

**Fig. 1:**
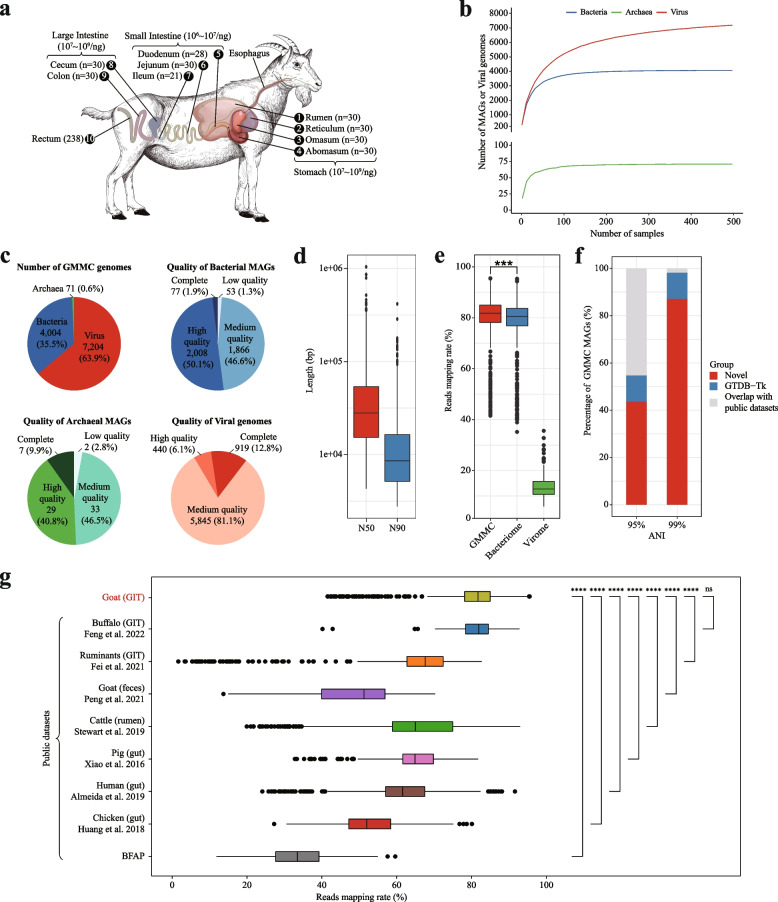
Reconstruction of the multi-kingdom microbial genomes of the goat gastrointestinal tract (GIT). **a** Sample collection along the goat GIT. A graphical representation of goat is shown with its GIT highlighted. The arrows along the GIT indicate the flow of food. The numbers in the parentheses next to the GIT site names indicate the samples obtained for this study. The GIT sites were divided into four sections in this study, namely stomach (rumen, reticulum, omasum, and abomasum), small intestine (duodenum, jejunum, and ileum), large intestine (cecum and colon) and rectum (fecal samples). The numbers beside the section names indicate the estimated numbers of microbes per nanogram DNA. **b** The rarefaction analysis of the unique number of bacterial/archaeal MAGs and viral genomes (*Y*-axis) as a function of sequenced samples (*X*-axis). The rarefaction curves for bacterial, archaeal, and viral genomes are shown in blue, green, and red, respectively (“Methods”). **c** Composition and quality of the genomes in the goat multi-kingdom microbiome catalog (GMMC), including 4004 bacterial and 71 archaeal MAGs and 7204 viral genomes. For GMMC MAGs, the quality criteria are defined by Bowers et al. [26]; complete: ≥ 90% completeness and ≤ 5% contamination according to CheckM [27] and at least 18 tRNA, high quality: ≥ 90% completeness and ≤ 5% contamination, medium quality: ≥ 80% completeness and ≤ 10% contamination, low quality: quality score (defined as the estimated completeness of a genome minus five times its estimated contamination) ≥ 50. For viral genomes, the quality is evaluated using CheckV [28]. **d** Contig N50 and N90 lengths (in bp) of GMMC genomes. **e** The mapping rates of clean reads to the GMMC genomes. The Wilcoxon rank sum test was used to show the statistical significance between groups; *** *P* < 0.001. **f** Percentages of novel bacterial and archaeal MAGs in GMMC as compared with public datasets at 95 and 99% average nucleotide identity (ANI) (“Methods”). **g** Mapping rates of metagenomic clean reads to the GMMC genomes as compared public datasets including the reference microbial genomes from the NCBI (BFAP, the combination of reference genomes including bacterial, fungal, archaeal, and protozoan reference genomes from the NCBI database) and MAGs of selected model organisms. The Wilcoxon rank sum test was used to show the statistical significance between groups; **** *P* < 0.0001)

In total, 497 samples were collected from 268 goats, including 259 intestine content samples from nine sites along the gastrointestinal tract (GIT) including rumen, reticulum, omasum, abomasum, duodenum, jejunum, ileum, cecum, and colon (Fig. 1a) and 238 rectum fecal samples (Table S1). The 259 content samples were taken from 30 slaughtered adult goats in Guangxi province, China (Table S1). The 238 rectum fecal samples were taken from the 238 live goats of three developmental stages (1, 6, and 12 months old; Table S3), two feeding styles (indoor feeding and grazing; Table S4), and four geo-locations (Yunnan, Sichuan, Guangxi, and Hainan provinces of China; Table S5). Of note, the nine GIT sites can be grouped into three broader sections, including stomach (rumen, reticulum, omasum, abomasum), small intestine (duodenum, jejunum, ileum), and large intestine (cecum, colon). Details of the samples are given in Table S1. All samples were immediately frozen after collection in liquid nitrogen and stored at − 80 °C until DNA extraction.

### DNA extraction, library construction, and metagenomics sequencing

Three grams of each sample was taken for DNA extraction. DNA was extracted by a bead-beating method using a mini-bead beater (Biospec Products; Bartlesville, USA), followed by phenol–chloroform extraction [29]. The total DNA was precipitated with ethanol, and the pellets were suspended in 50 μL of Tris–EDTA buffer (Vazyme, Nanjing, China). DNA was quantified using a NanoPhotometer® (IMPLEN, CA, USA) following staining using a Qubit® 2.0 Flurometer (Life Technologies, CA, USA). DNA samples were stored at − 80 °C until further processing.

Library preparation was performed according to the TruSeq DNA Sample Preparation Guide (Illumina, 15,026,486 Rev. C) method and procedure using 500 ng DNA as template. Qualified libraries were selected and subjected to the Illumina NovaSeq 6000 for pair-ended sequencing with read length of 150 base pairs (PE150).

### Estimation of numbers of microbes in different GIT sections

We adopted a method to estimate the numbers of each bacteria cell in different GIT sections of goat based on qPCR using a standard curve. Briefly, to construct a standard curve for the bacteria, we cloned a conserved region (27f/1492r) [30] of the 16S rRNA gene using Q5 High-Fidelity DNA Polymerase (New England Biolabs, Massachusetts, USA) according to the reported primers (16S clone primer: forward-AGAGTTTGATCCTGGCTCAG reverse-TACGGCTACCTTGTTACGACTT) [31]. Cloned fragments were purified by OMEGA Gel Extraction Kit (Omega Bio-Tek, USA) and ligated to pEASY-Blunt simple vector (TransGen Biotech, Beijing, China; M13 primer: forward-TGTAAAACGACGGCCAGT reverse-CAGGAAACAGCTATGACC).

A standard curve and an equation were then generated by linear regression, as detailed below:

After ligating the 16S sequence into the pEASY-Blunt vector, we transformed *Escherichia coli* and selected positive clones. Positive transformants were identified by Sanger sequencing. Subsequently, we cultured *E. coli* harboring the positive plasmids and extracted the pEASY-16S plasmids using a kit (Endo-Free Plasmid Mini Kit I D6948, OMEGA). After a serial dilution (twofold) of the plasmids, PCR amplification was performed to obtain the CT value. The copy number was then calculated using the formula:

$$Sample\;concentration\;(ng/\mu l) \times 10 - 9 \times 6.02 \times 1023/bp \times 660=copies/\mu l$$

By calculating the copy number, we plotted the CT value on the *x*-axis and the logarithm of copies/μl on the *y*-axis, fitting a standard curve. The equation of the fitted standard curve was:

$$y= -0.3408x+12.079\;with\;an\;R2=0.9961$$

When the R^2^ (amplification efficiency = 2, standard curve equation is: *y* =  − 0.3408x + 12.079) of the standard curve (Fig. S10) is more than 0.99, it will be considered acceptable for quantitative analysis (Bacteria quantification primer [32]: forward-ACTCCTACGGGAGGCAG reverse-GACTACCAGGGTATCTAATCC).

The DNA extracted from the GIT samples were used as templates for qPCR analysis. Real-time PCR was performed using the7500 Real-Time PCR System (Applied Biosystems, USA) detection system with fluorescence detection of SYBR green dye. Components of qPCR included the 16S forward and reverse primers mentioned above (10um/ul), 50 ng DNA samples, 2 × AceQqPCR·SYBR Green Master Mix 10ul (Vazyme, Nanjing, China) and DNase-free water to 20 μ L for 40 cycles (denaturation at 95℃ for 30 s, annealing at 60℃ for 15 s, and extension at 72℃ for 35 s, with a total of 40 cycles). The CT values of the bacteria in the samples were detected by qPCR, and the bacterial copy numbers were converted by substituting into the standard curve equation.

Please consult Table S9 for the resulting equation of the standard curve, the CT values, and numbers of bacteria in each GIT section.

### Quality control and removal host- and food-associated genomes

We submitted all samples for pair-end metagenomic and obtained a total of ~ 3 Tb of raw reads. Raw reads were trimmed by Trimmomatic (v 0.39) [33] with the options “ILLUMINACLIP: TruSeq2-PE. fa:2:30:10 SLIDINGWINDOW:15:30 MINLEN:110 TRAILING:30 AVGQUAL:30,” followed by removal of reads that could be aligned to the host (*Capra hircus*, GCF_001704415.1) [34] or food (*Zea mays*, GCF_000005005.2; *Medicago truncatula*, GCF_000219495.3 and *Glycine max*, GCF_000004515.5) [35–37] genomes using Bowtie2 (v 2.3.5.1) [38]. Overall, a median of ~ 1.3G bases were removed from each sample. The remaining paired “clean reads” were then used for further analyses.

### Generation and quality assessment of metagenome-assembled genomes (MAGs)

Unless otherwise stated, default parameters were used for metagenome assembly. In brief, each sample was assembled using metaSPAdes (v 3.13.0) [39] with the options “-k 35,45,55,65,75,85,95,105 -t 20 -m 180” and MEGAHIT (v 1.2.8) [40]. Co-assemblies were also performed on combined samples according to their respective groups (i.e., samples of the same GIT site, age, feeding style, or geo-location) by using MEGAHIT (v 1.2.8) [40].

BWA-MEM (v 0.7.17) [41] was used to map clean reads back to the filtered assembly, and Samtools (v 1.9) [42] was used to convert the aligned results to BAM format. A script “jgi_summarize_bam_contig_depths” from the MetaBAT2 (v 2.12.1) [43] package was used to calculate coverage from the resulting BAM files. Metagenomic binning was applied to both the single-sample assemblies and the co-assemblies using MetaBAT2. The single-sample assembly binning produced a total of 24,122 bins, and the co-assembly binning produced a further 18,588 bins. All 42,710 bins were combined and dereplicated using dRep (v 2.3.2) [44]. The dRep dereplication workflow was used with options “dereplicate -nc 0.1 -p 20 -comp 80 -con 10 -str 100 -strW 0,” and this workflow also requires HMMER (v 3.3) [45], PRODIGAL (v 2.6.3) [46], pplacer (v 1.1.alpha19) [47], ANIcalculator (v 1) [48], MUMmer (v 3) [49], and Centrifuge (v 1.0.4) [50]. In prefiltering, bins assessed by CheckM (v 1.1.1) [27] as having both completeness ≥ 80% and contamination ≤ 10% were retained for pairwise dereplication comparison. Only the highest scoring MAG from each secondary cluster was retained in the dereplicated set. For our dataset, 4075 dereplicated MAGs were obtained.

### Identification and quality evaluation of viral genomes

Viral genomes were identified by a bioinformatics pipeline similar to Luis et al. [51]. Briefly, after assembly, contigs of ≥ 1.5 kb were used to identify viral sequences using VirSorter2 (v 2.1) [52] with the options ‘–include-groups "dsDNAphage, ssDNA" –min-score 0.7’ and VirFinder (v 1.1) [53] with default parameters. Contigs were identified as viruses by both VirSorter2 (v 2.1) [52] and VirFinder (v 1.1) [53] (score ≥ 0.6 and *p* < 0.05). The completeness of the viral contigs was estimated using CheckV (v 0.8.1) [28]; 12,355 viral contigs with > 50% completeness were clustered into species-level viruses operational taxonomic units (vOTUs) on the basis of 95% ANI and 85% alignment fraction (AF) of the shorter sequence similar to Nayfach S et al. [54]. In total, 7204 putative viral genomes of length > 5kb were identified.

### Taxonomic assignments of the MAGs and viral genomes

Taxonomic assignments of the 4075 bacterial/archaeal MAGs were performed using the GTDB-Tk (v 1.2.0) [55] using the “classify_wf” workflow. The results were visualized in GraPhlAn (v 1.1.3) [56] as a phylogenetic tree.

To taxonomically classify the viral genomes, VirusTaxo (https://github.com/omics-lab/VirusTaxo, downloaded at 19th April, 2022) [57] was used to compare the nucleotide sequences against those in the prebuilt database of VirusTaxo and assign a genome to a known viral genus at an entropy index threshold of < 0.5. A Demovir script (https://github.com/feargalr/Demovir; downloaded at 6th January, 2022) was then used to predict family and order ranks for the remaining genomes by searching for viral marker genes at the amino acid level.

We referred the 4004 bacterial and 71 archaeal MAGs and 7204 viral genomes as to the goat multi-kingdom microbiome catalog (GMMC).

### Mapping clean reads to GMMC and reference microbial genomes from selected model organisms

To show the GMMC genomes could improve the coverage of goat microbial reads, public microbial genome datasets were first downloaded, including the MAGs from the buffalo GIT [10], ruminants GIT [9], goat feces [24], cattle rumen [22], pig gut [58], human gut [23], chicken gut [59], and a set of genomes combined from the bacterial, archaeal, fungal, and protozoan reference genomes from the NCBI RefSeq [60] (referred as to BFAP). Then BWA-MEM (v 0.7.17) [41] was used to map the clean reads to these public datasets and the GMMC genomes. A mapping rate was calculated for each sample as the percentage of clean reads mapped to each of the datasets by Samtools (v 1.9) [42] with the “flagstat” option.

### Comparing the GMMC genomes with the sequences in public datasets

To reveal the novelty of the GMMC bacterial and archaeal MAGs, fastANI (v 1.1) [61] was used to calculate the ANI between the GMMC MAGs and the sequences in the abovementioned databases. Different ANI thresholds were used in this study, including 95 and 99%.

The fastANI tool (v 1.1) [61] was also used to calculate the ANI between the GMMC viral genomes and the sequences in several public viral databases including the Gut Virome Database (GVD) [62], Metagenomic Gut Virus (MGV) [54], Gut Phage Database (GPD) [51], and NCBI viral Reference genomes, Release 201 (Fig. S2a, downloaded at 6th July, 2020). Different ANI thresholds were used in this study, including 95 and 99%.

### Gene annotation and functional characterization of non-redundant proteins

All contigs were annotated for protein-coding genes using MetaGeneMark (v 3.38) [63] and Prokka (v 1.14.5) [64] with the options “–metagenome –kingdom Bacteria –force –norrna –notrna –cpus 20.” A total of 4,817,256 non-redundant protein-coding genes were obtained by CD-HIT (v.4.8.1) [65] with the option “-c 0.95 -aS 0.90.” Salmon (v 0.10.1) [66] was used to estimate the coverage of genes.

To annotate these genes, HMMER (v 3.3) [45] was used to compare the protein sequences with those in the Carbohydrate-Active Enzymes database (CAZymes) [67] with default parameters and a threshold of *p* < 0.05. eggNOG-mapper (v 0.12.7) was also used to annotate these genes against the eggNOG database (v 5.0) [68].

tRNA genes were annotated using tRNAscan-SE (v 2.0) [69], and 16S rRNA genes were predicted using barrnap (https://github.com/tseemann/barrnap, v 0.9).

### Calculation of the relative abundance for GMMC genomes

To calculate the relative abundance of each GMMC genome, clean reads of each sample were mapped to the GMMC genomes using BWA-MEM (v 0.7.17) [41] with default parameters. After converting the resulted SAM files to BAM format using Samtools (v 1.9) [42], the coverage of each genome was determined. An in-house R script was used to calculated transcripts per million (TPM) for each genome. The relative abundances for higher taxonomic levels such as genus, family, and order were also determined by summing up the abundances of their daughter clades according to the phylogenetic tree provided by GTDB-Tk.

### Rarefaction analyses of the GMMC genomes and non-redundant protein-coding genes

An in-house R script was used to perform the rarefaction analyses [70] for the bacterial, archaeal, and viral genomes and the non-redundant protein-coding genes, respectively. Briefly, a threshold of TPM > 100 was used to determine whether a genome or gene was present in a sample. *n* samples were randomly selected from the 497 samples and the unique genomes and genes were counted; here *n* ranged from 1 to 497 and the sampling for each *n* was repeated 100 times. Then the numbers of unique genomes and genes were plotted as a function of the sample size (i.e., *n* using a R package ggplot2 [71].

### Analysis of microbial diversity between sample groups and effects of host and environmental factors

To calculate and visualize differences among groups (e.g., different GIT sites, ages, feeding styles, and geographical locations), a non-metric multidimensional scaling (NMDS) method and ANOSIM analysis were used to compare the microbial diversities between groups [18, 59]. They both used the relative abundance profiles of the GMMC genomes and were implemented in the R package “vegan” (v 2.5.7) [72].

The permutational multivariate analysis of variance (PERMANOVA) [73] implemented in the R package “vegan” was used to determine the impacts of various host and environmental factors to the microbial diversities of the GMMC genomes (single- and multiple-factor analysis), including the GIT site, age, feeding style, and geography.

### Identification of differentially abundant taxa between groups

The linear discriminant analysis (LDA) implemented in the LEfSe tool [74] was used to identify differential taxa between groups of samples. The LDA score > 2 and *p* < 0.05 were used as the cutoff for selecting the differential taxa. Wilcoxon test was used to validate the statistical significance in the relative abundances (TPMs) of the differential taxa between groups.

### ***Trend analysis of F/B ratio***

To identify trend clusters of the F/B ratio (*Firmicutes_all* to *Bacteroidota*, *Firmicutes_all* is the combination of *Firmicutes*, *Firmicutes_A*, *Firmicutes_B* and *Firmicutes_C,* also called *Bacillota*) along the goat GIT sites, a R package Mfuzz [75] was used to cluster the 23 goats that at least had samples from seven out of nine GIT sites. Two clusters were obtained that could clearly separate the F/B ratios.

### Lifestyle and host analysis of the GMMC viruses

DeePhage (v 1.0) [76] was used to predict the lifestyles of the GMMC viruses. According to the DeePhage score, the viruses were classified as virulent/lytic (score ≥ 50) or temperate (score < 50). To predict viral-host relationships between the 7204 viral and 4075 bacterial/archaeal GMMC genomes, the following four methods were used.

1. CRISPR-spacer matches. CRISPR spacers of the bacterial/archaeal GMMC genomes were identified using CRT (v 1.2) [77] and MinCED (v 0.4.2, https://github.com/ctSkennerton/minced). The union of the CRISPR spacers was then aligned to the GMMC viral genomes using blastn (v 2.5.0) [78] with options of “-word_size 10 -dust no -max_target_seqs 10,000.” Matches with mismatch ≤ 1 and alignment length > 95% spacer length were retained.
2. Nucleotide sequence similarity searches. Blastn was used to compare the GMMC viral and bacterial/archaeal genomes. A putative viral-host relationship could be established if their nucleotide sequences shared > 90% identity over > 500 bp similar to Nayfach et al. [79].
3. Binning results. A viral-host relationship could also be established if the viral contig was binned into the MAGs based on the MetaBAT2 binning results.
4. k-mer similarities. A VirHostMatcher (v 1.0.0) [80] tool by default parameters (k-mer length = 6bp) was used to predict the viral-host relationships based on k-mer similarities between the viral and host genomes. A virus was predicted to have host relationship with MAGs if the VirHostMatcher score ≤ 0.25; up to five hosts would be taken from the predicted results.

### Statistics

In addition to the aforementioned software, we utilized R packages ggplot2 [81], UpSetR [82], pheatmap [83], and ggpubr [84]; and adjusted the phylogenetic trees by using the itol [85].

Unless otherwise specified, the Wilcoxon rank sum test model was used to show the statistical significance between groups, and the statistical data were derived from 497 goat gut microbiome sequencing data obtained through our sequencing.

## Results

### Construction of the goat multi-kingdom microbiome catalog (GMMC)

To provide a comprehensive overview of the microbes associated with the gastrointestinal tract (GIT) of goat, we collected a total of 497 samples (Table S1) from ten GIT sites, including 259 content samples from nine GIT sites in three sections, namely stomach (rumen, reticulum, omasum, abomasum), small intestine (duodenum, jejunum, ileum), and large intestine (cecum, colon; Table S2), and 238 rectum fecal samples that spanned three developmental stages (1-, 6-, and 12-month old; Table S3), two feeding styles (indoor feeding and grazing; Table S4), and four geographical locations (Yunnan, Sichuan, Guangxi and Hainan provinces of China; Table S5). We estimated the numbers of each bacteria cell were 10^7^ ~ 10^9^/g 10^6^ ~ 10^7^/g and 10^7^ ~ 10^9^/g for the stomach, small intestine, and large intestine, respectively (Fig. 1a).

After removing vector sequences, low-quality bases, short reads, and the host and food genomes, we obtained in total 2.7 Tb clean data with on average 36,730,204 reads and 5,485,416,272 bases per sample (Table S6). We assembled the clean reads and grouped the obtained contigs into a total of 42,710 bins (also known as metagenome-assembled genomes, MAGs). Then we dereplicated the MAGs at an ANI of 99%, filtered out those of ≤ 80% completeness or contamination of ≥ 10%, and obtained a total of 4,075 MAGs longer than 200kb. We annotated them using GTDB-Tk [55] and identified a total of 4004 bacterial and 71 archaeal MAGs (Table S7). Among which, ~ 47% were of high-quality with completeness > 90% and contamination < 5% (Fig. 1c) according to the criteria defined by Bowers et al*.* [86], and nine were 100% completeness (Table S7). 97.89% of the MAGs contained multiple tRNA genes (tRNA type number ≥ 10, Table S7) with each contained ~ 15.9 tRNA types. However, only 83 MAGs contained one or more full-length 16S rRNA gene (Table S7) and 276 MAGs encoded partial 16S rRNA genes, likely because that the short-read assemblies could not assemble such highly similar regions. Overall, the 4075 MAGs were on average 2.17Mb in size (0.61 Mb ~ 7.13Mb; Table S7) with a mean N50 length (the sequence length of the shortest contig at 50% of each MAG total length; Fig. 1d) of 43.97kb (4.30kb ~ 1.04Mb; Table S7); they encoded 494 to 5897 protein-coding genes with a mean of 1,802 (Table S7). In addition, we also annotated a total of 12,355 putative viral contigs (mostly bacteriophages) using a bioinformatics pipeline similar to Luis et al. [51] and dereplicated them into 7204 non-redundant viral genomes at an ANI of 95% with a minimal length of 5kb. Among which, 18.86% were of high-quality with completeness > 90% according to a CheckV tool [28]. Interestingly, we did not identify any eukaryotic genomes using methods similar to Paul et al. [87], suggesting that either the eukaryotic genomes were very rare in our samples or our methods were not optimized for recovering these genomes. Rarefaction analysis results suggested that we had collected enough samples for recovering bacterial and archaeal genomes in the goat GIT, as indicated by Fig. 1b and Fig. S5b that the numbers of non-redundant bacterial, archaeal, and viral genomes plateaued around 100 samples. Together, we referred the 4004 bacterial and 71 archaeal MAGs and 7204 viral genomes as the goat multi-kingdom microbiome catalog (GMMC).

To check whether the GMMC genomes could improve the coverage of goat GIT associated microbial genomes, we used them to recruit the clean sequencing reads and found that 81.8% of the reads could be mapped to the GMMC genomes, including 80.5 and 12.8% could be mapped to the bacterial/archaeal MAGs and viral genomes, respectively (Fig. 1e). The overall read mapping rate was significantly higher than most public microbial genome databases including those from the ruminants GIT [9], goat feces [24], cattle rumen [22], pig gut [58], human gut [23], chicken gut [59], and a combined dataset of bacterial, fungal, archaeal, and protozoan reference genomes from the NCBI database [60] (BFAP, Methods; Fig. 1g).

We then analyzed the novelty of the GMMC genomes by comparing them with the sequences in the abovementioned public datasets and also annotating using the GTDB-Tk [55]. At ANI thresholds of 95 and 99%, 43.71% (*n* = 1781) and 87.21% (*n* = 3554) of the GMMC bacterial/archaeal MAGs were novel respectively (i.e., they did not have nucleotide identities above the thresholds with sequences in any of the public database including GTDB and those used in Fig. 1g; in addition, at the same ANI thresholds, 90.91% (*n* = 6549) and 96.23% (*n* = 6933) of the viral genomes were novel as compared with several public viral databases including the Gut Virome Database (GVD) [62], the Metagenomic Gut Virus (MGV) [54], the Gut Phage Database (GPD) [51], and NCBI viral Reference genomes, Release 201 (Fig. S2a, downloaded at 6th July, 2020).

In summary, we assembled a GMMC catalog including 4004 bacterial and 71 archaeal MAGs and 7204 viral genomes that better represented the goat gastrointestinal microbiota while contained a significant proportion of novel genomes.

#### Taxonomic and functional annotation of the GMMC genomes

**Fig. 2:**
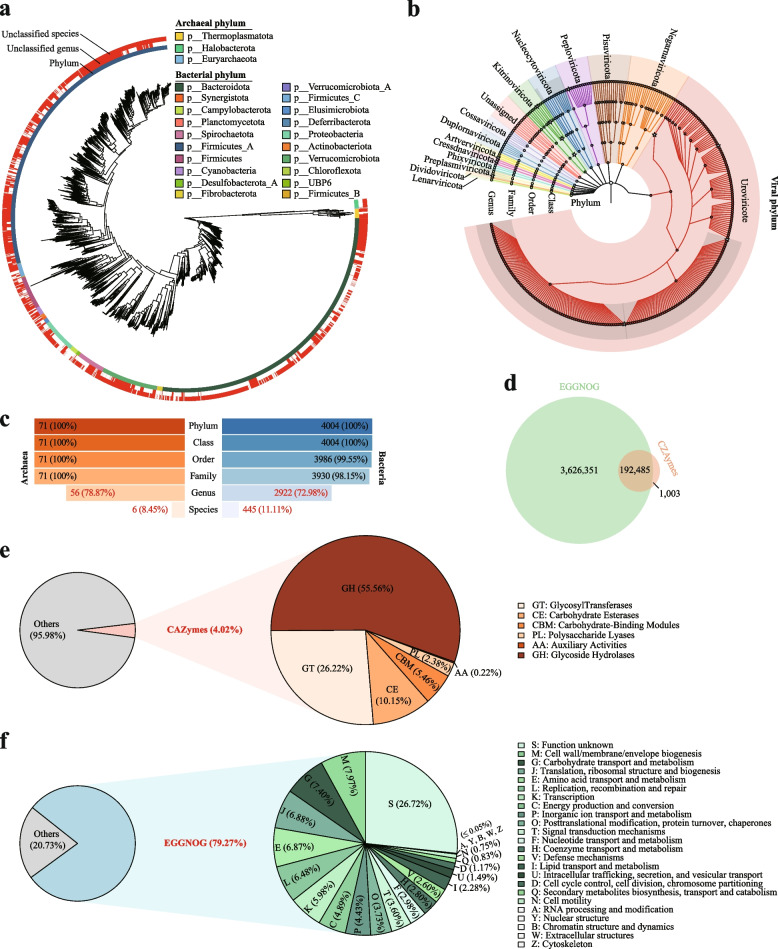
Taxonomic and functional annotation of the GMMC genomes. **a** The phylogenetic relationship among the 4004 bacterial and 71 archaeal MAGs in the GMMC and their taxonomic classification according to GTDB-Tk [20]. The annotations from inside to outside represent annotations of species level (different colors represent different phyla), unclassified genus (in red), and unclassified species (in red). **b** Taxonomic among the 7204 viral genomes, color-coded by the viral phyla. The stars at the internal and leaf branches indicate unclassified rank by VirusTaxo and Demovir. **c** Classification rates of bacterial (right) and archaeal (left) MAGs in GTDB at different taxonomic levels. **d–f** Annotations of the non-redundant proteins using the CAZymes (**e**) and eggNOG (**f**) databases; Venn diagram (**d**) shows the overlap of the annotated protein-coding genes between eggNOG (green) and CAZyme (Orange); pies show the proportions of proteins annotated by these two methods (left) and the overall categories (right)

We first assigned the taxonomic classifications to the bacterial and archaeal MAGs in the GMMC using GTDB-Tk [55]. Of the 4075 MAGs, all could be assigned to their respective kingdoms (bacterial, *n* = 4004; archaeal, *n* = 71) and most could be assigned to known taxonomy at the phylum, class, order, and family levels (Fig. 2a, c). However, at the species level, only 451 (11.07%) of the MAGs could be classified as known species (Fig. 2c), indicating most the MAGs were previously unidentified (i.e., not present in the GTDB database), consistent with our analysis using the ANI (Fig. 1f). At the phylum level, the bacterial MAGs were dominated by *Firmicutes_A* (*n* = 1503) and *Bacteroidota* (*n* = 1479), followed by *Verrucomicrobiota*, *Proteobacteria*, and *Spirochaetota*. All members of *Firmicutes_A* belonged to the class *Clostridia*, which included the orders *Oscillospirales* (*n* = 867), *4C28d-15* (*n* = 281), *Lachnospirales* (*n* = 236), and *Lachnospiraceae* (*n* = 225)*.* All members of *Bacteroidota* belonged to the class *Bacteroidia*, which included the orders *Bacteroidales* (*n* = 1474) and *Flavobacteriales* (*n* = 5: containing only the family *UBA1820*). Species in the order *Verrucomicrobiota* were divided into three classes, *Lentisphaeria* (*n* = 180), *Verrucomicrobiae* (*n* = 61), and *Kiritimatiellae* (*n* = 45). All archaeal MAGs were known methane producers belonging to the phylum *Thermoplasmatota* (*n* = 37), *Halobacterota* (*n* = 30), and *Euryarchaeota* (*n* = 4). It is noteworthy that our prior buffalo study revealed a greater count of Halobacterota MAGs (*n* = 84) compared to Euryarchaeota (*n* = 24) [88]. These findings suggest higher strain diversity within Halobacterota relative to Euryarchaeota among ruminants. Additionally, in terms of relative abundances, we observed Halobacterota MAGs in only 48.89% (243/497) of the goat samples, while Euryarchaeota MAGs were present in 90.34% (449/497) of goat samples. This further reinforces the prevailing notion of Methanobrevibacter is a widely prevalent and important classification in ruminant animals [89].

A ruminant GIT bacterial/archaeal genome catalog (the Ruminant Catalog hereafter) was recently constructed from multiple organisms including six goats [9]; we thus also compared this catalog with our GMMC MAGs in more details. Overall, 56.37% (*n* = 2297) of the GMMC MAGs had ANI < 95% with those in the Ruminant Catalog (Fig. S3a; Table S7); among these, 1241 were *Firmicutes* (*Firmicutes* and *Firmicutes_A*) and mainly from the orders of *Oscillospirales* (53.26% out of the 1241 MAGs), *4C28d-15* (14.75%), and *Lachnospirales* (14.18%) (Fig. S3b). Importantly, species in the orders *of Oscillospirales* and *Lachnospirales* are known to have digestive functions [90, 91], while *4C28d-15* is known to be abundant in the rumen [92]. These results suggested that our GMMC MAGs could significantly expand the public databases with functionally important microbial taxa.

We next annotated the GMMC viral genomes by using VirusTaxo [57] and Demovir tools (https://github.com/feargalr/Demovir; downloaded at 6th January, 2022). And we assigned 75.67% of them to known taxonomical clades (Fig. 2b, S4); among which, 602 viruses were assigned to the family of Poxviridae, followed by Mimiviridae (*n* = 573), Microviridae (*n* = 477), and Siphoviridae (*n* = 120). The overall taxonomic distribution was similar to the other metagenome-derived viral catalogs in humans including the GVD, MGV, and GPD.

We annotated a total of 7,645,971 protein-coding genes from all the GMMC genomes and dereplicated them into a non-redundant set of 4,817,256 genes at a 95% amino-acid similarity threshold using CD-HIT [65]. Rarefaction analysis showed that the numbers of genes plateaued at ~ 150 samples, suggesting our samples were sufficient for recovering most of these genes; similar trends were found for both the content and fecal samples (Fig. S5a). We queried their protein sequences against popular databases and annotated 79.27% (58.09% were assigned to known functions; Fig. 2d, e, f) and 4.02% of them according to the eggNOG [68] and CAZyme [67] databases, respectively. Together, 20.70% (*n* = 997,417) of the genes had no homologs in public protein databases and 48.05% were not assigned to known functions by either database, suggesting that almost half of the proteins may code for novel functions.

#### Microbial community dynamics along goat GIT were driven by diet and associated with functional burdens and disease risks of GIT sites

**Fig. 3:**
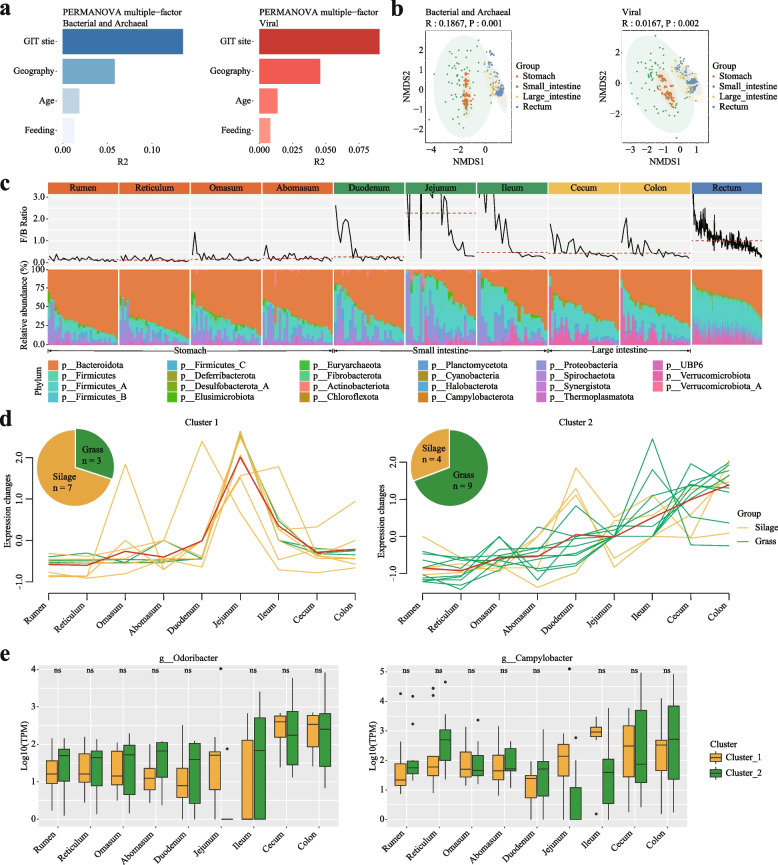
Microbial community dynamics along goat GIT and the influence of diet. **a** Factors contributed significantly to the overall microbial community compositions as determined by multiple-factor analysis results using bacterial and archaeal (left) and viral (right). Horizontal bars represent the amount of inferred variance (adjusted R2) explained by each identified covariate. All factors were found to be significantly associated with gut microbial variations (*P* = 0.001). **b** Non-metric multidimensional scaling (NMDS) analysis based on between-sample Bray–Curtis dissimilarities shows the relationships among the samples from the four goat GIT sections. **c** Overall *Firmicutes_all* to *Bacteroidota* (F/B) ratios (top) and the detailed relative abundances of top phyla (bottom) in the goat samples, grouped according to their GIT sites and sections. **d** Distinctive trends of F/B ratios along the GIT sites revealed by clustering analysis and the impact of feed types; each line represent a goat that had samples from more than seven out of nine GIT sites, color-coded according to different diets (green: grass feed, yellow: silage feed), with the red line representing the inferred trend. The pie chart shows the proportions of the dietary groups of the goat in the corresponding cluster. **e** Relative abundances of genus in two F/B clusters with goat GIT sites. The relative abundances were calculated as reads count per million sequenced clean reads (TPM, log10 transformed; “Methods”). The Wilcoxon rank sum test was used to show the statistical significance between groups. ns: no significance, * *P* < 0.05, ** *P* < 0.01, *** *P* < 0.0001, **** *P* < 0.0001

We next evaluated factors influencing the goat GIT microbiota at the community composition level (i.e., the members and their relative abundances of a community) [93], including the GIT site, geography, host age, and feeding style. We observed that the GIT site exerted the strongest effect, followed by geography, age, and feeding style using both the single- and multiple-factor permutational multivariate analysis of variance using PERMANOVA (“Methods,” *P* < 0.001; Fig. 3a); we obtained similar trends using both methods and on both the bacterial/archaeal and viral genomes.

To examine whether samples from the same GIT section, i.e., stomach, small intestine, large intestine, and rectum (fecal samples), could have similar community compositions, we performed a non-metric multidimensional scaling (NMDS) analysis on between-sample dissimilarities (Bray–Curtis) using the relative abundances of the GMMC MAGs as input. We found significant clustering according to the GIT site (analysis of similarities (ANOSIM); bacterial/archaeal: *R* = 0.1867, *P* < 0.001; viral: *R* = 0.0167, *P* < 0.002; Fig. 3b). As shown in Fig. 3b, samples from the large intestine and rectum clustered together and were separated from those from the stomach, which was expected. These results suggested distinctive microbial compositions at different GIT sections.

We then examined the microbial dynamics along the goat GIT in more details. Overall, *Firmicutes_all* (i.e., the combination of *Firmicutes*, *Firmicutes_A*, *Firmicutes_B* and *Firmicutes_C,* also called *Bacillota*) and *Bacteroidota* were the two most abundant phyla, accounting for 51.27 and 25.82% of total relative abundances, respectively (Fig. 3c). Despite significant between-sample variations in the microbial compositions, we observed that the *Firmicutes_all* to *Bacteroidota* ratios (F/B ratios) of the stomach samples remained mostly constant and were comparable within and between the four stomach chambers (i.e., rumen, reticulum, omasum, abomasum) (Fig. 3c). The F/B ratios started to show significant within-site fluctuations from the small intestine (Jejunum) and further to the downstream GIT sites (Fig. 3c). To explore the underlying contributing factors, we applied a de novo clustering analysis on the F/B ratio dynamics along the goat GIT and obtained two clusters using 23 goats that at least had samples from seven out of nine GIT sites (Fig. 3d). The cluster 1 showed relatively low and comparable F/B ratios in the stomach and large intestine, with a sudden peak in the small intestine, especially the jejunum and ileum. Conversely, cluster 2 showed a steady increase of the F/B ratio along the goat GIT (Fig. 3d). We compared the diets of the two groups and found that most of the goats in the cluster 1 were fed with silage whereas most goats of cluster 2 were fed with grass (Fig. 3d and S6). Previous studies suggested that the *Firmicutes_all* and *Bacteroidota* species represented digestive versus absorptive capacities [94, 95]; therefore, the F/B ratio dynamics indicated different digestion/absorption burdens along the goat GIT sites, i.e., in this case, the fermented silage was absorbed in the small intestine in advance, whereas the grass feed was gradually absorbed along the intestinal tract of goats. Our results thus were consistent with the fact that the silage feed was easier to digest [96] so that only a few GIT sites were involved in the digestive process (hence the high F/B ratios), whereas the grass feed was harder to digest and required more GIT sites to be involved.

The two F/B ratio trends along the goat GIT were also associated with distinctive disease risks. We compared the relative abundance of different genera in the two clusters along the GIT sites and found that the relative abundance of *Odoribacter* and *Campylobacter* in cluster 1 at jejunum increased significantly (Fig. 3e). *Campylobacter* is a landmark genus causing enteritis [97], indicating increased disease risks. Conversely, the *Odoribacter* has been shown to able to effectively limit intestinal inflammation [98], this indicates that the microbiota in the jejunal digestion site of goats has the potential role of resisting intestinal inflammation.

#### Distribution and variation of microbial taxa along the goat GIT

**Fig. 4:**
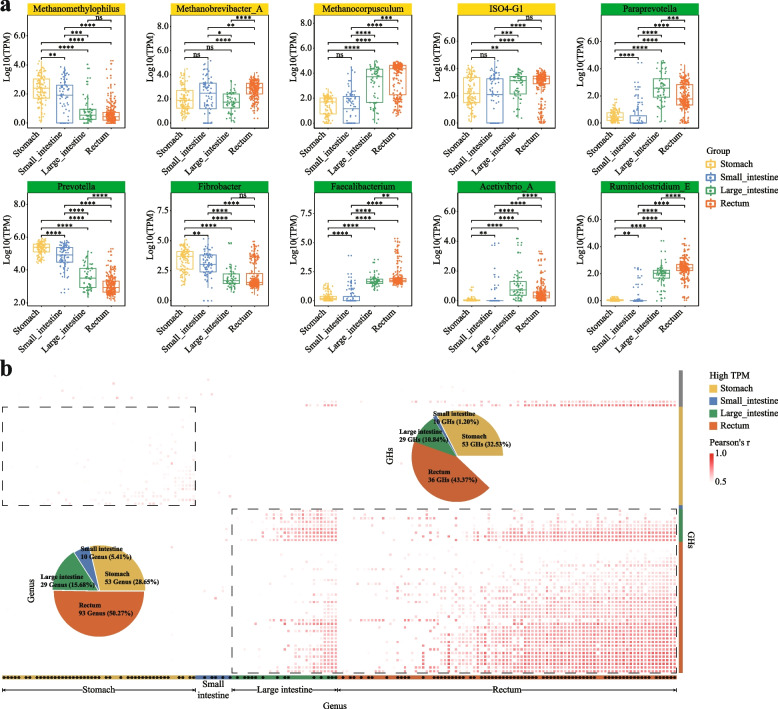
Distribution and variation of functionally important genera along the goat GIT. **a** Variation of methane-producing (with yellow headers) and cellulose-digestive (with green headers) genera along the goat GIT; their relative abundances were calculated as reads count per million sequenced clean reads (TPM, log10 transformed; “Methods”). Their differential distribution was identified between groups by using LEfSe (see “Methods”); the Wilcoxon rank sum test was used to show the statistical significance between groups. ns: no significance, * *P* < 0.05, ** *P* < 0.01, *** *P* < 0.0001, **** *P* < 0.0001. **b** Genera whose relative abundances showed significant positive correlations with those of the CZAyme categories involved in cellulose digestions in different GIT sites. The heatmap color indicates the correlation coefficients (*r*); significant positive correlation results with *r* $\ge$ 0.5, and *P* < 0.05 was retained. Pie chart shows the GIT sites in which the genera showed the highest relative abundance and GHs classification. * indicates the genera that were not previously annotated to have cellulose digestive capabilities

We next explored the distribution and variation in the annotated microbial taxa along the goat GIT in more details, especially those with known functions in methane production and cellulose digestion [99, 100]. We identified a total of 311 genera that showed significant abundance variations among the four GIT sections (two-group Wilcoxon rank sum test, *p* < 0.05). Among which, all four methane-producing genera were differential distributed, including *Methanomethylophilus*, *Methanocorpusculum*, *Methanobrevibacter_A* and *ISO4-G1* (Fig. 4a). Interestingly, *Methanomethylophilus* was the only genus that showed the highest relative abundance in the stomach group than other sections, whereas the other genera showed either increasing abundances from stomach (lowest) to rectum (highest) such as the *Methanocorpusculum*, or varied distributions along the GIT such as the *Methanobrevibacter_A* and *ISO4-G1* (Fig. 4a; see also Fig. S7 for their trends in the individual GIT sites). These results suggested that GIT sections other than the stomach were also involved in methane production, consistent with our previous observations in the buffalo GIT microbiota [10].

We also observed significant variations in the annotated cellulose-digestive bacteria, including six genera of *Prevotella*, *Fibrobacter*, *Faecalibacterium*, *Acetivibrio_A*, *Ruminiclostridium_E*, and *Paraprevotella*. The first two genera, i.e., *Prevotella* and *Fibrobacter*, showed higher relative abundance in the stomach and small intestine, whereas the other four genera showed higher abundances in the downstream sections (i.e., large intestine and rectum; Figs. 4a and S7). These functional bacterial genera reflect the trend of host-microbe interactions in goats, where they collectively contribute to digestion and absorption functions across different gastrointestinal locations. For example, it has been reported that the *Prevotella* species played a pivotal role in hemicellulose digestion and were involved in starch, cellulose, hemicellulose, and pectin conversion in Bovine rumen [101], whereas the *Fibrobacter* species were involved in cellulose digestion with *Prevotella* species together [101, 102].

Because the cellulose digestive capabilities of the GIT microbiome were essential to the goat development and qualities, we next set out to identify novel genera that have putative cellulose digestive functions. We correlated the abundances of the CZAyme categories involved in the glycoside hydrolases (GHs), i.e., enzymes related to cellulose digestion [103] with those of the individual genus. We identified 185 genera (out of the 311 differential taxa) that showed significant positive correlations with at least one GHs category (Pearson correlation coefficient R ≥ 0.5, *P* < 0.05). We found that 49 out of the 185 genera (26.5%) were reported to have cellulose digestive capabilities, covering all such genera that we annotated in the GMMC (Table S7), supporting the validity of our methods; the remaining 136 genera (73.5% out of 185) thus were worth further investigated. Surprisingly, 65.95% of these genera had higher relative abundance in the sections of the large intestine and rectum (15.68% in the large intestine group, 50.27% in the rectum group), whereas only 28.65% had higher relative abundance in the stomach, supporting the important role of the downstream GIT sections (i.e., large intestine and rectum group) in cellulose digestion. Further work is needed to experimentally validate the capacities of these genera and their substrate specificities.

#### Variation of goat GIT microbiota associated with age, feeding style and geography

**Fig. 5:**
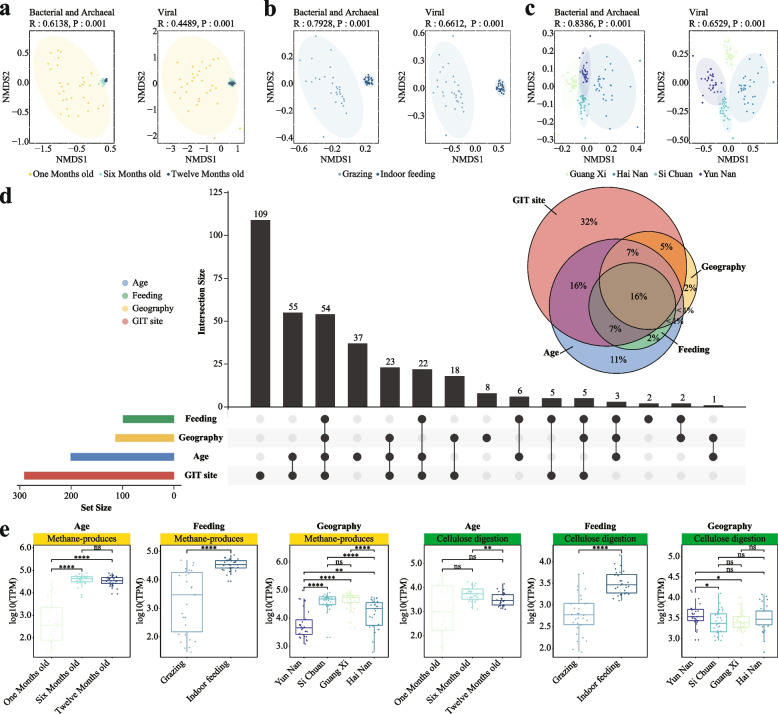
Variation of goat GIT microbiota associated with age, feeding style, and geography.** a**–**c** Non-metric multidimensional scaling (NMDS) based on Bray–Curtis dissimilarities (“Methods,” subsets (samples at line 262–499) as indicated in Table S1) show significant clustering of samples according to the goat age (**a**), feeding style (**b**), and geography (**c**), respectively. **d** The Venn and upset diagram shows the shared and unique relationships among differentially abundant genera across various factors (including the age, feeding style, geography, and GIT sections), the genera shown in the figure are all genera that exhibit differences in at least one factor. **e** Variation of the methane production and cellulose digestion-related genera attributed to different age, feeding style, and geography groups. Yellow and green represent methane production and cellulose digestion-related genera, respectively. Differential taxa were identified between two groups using linear discriminant analysis effect size (LEfSe) analysis (“Methods”); the Wilcoxon rank sum test was used to test the statistical significance between groups. ns: no significance, * *P* < 0.05, ** *P* < 0.01, *** *P* < 0.0001, **** *P* < 0.0001

Factors other than the GIT site also significantly affected the overall microbial community compositions of goat GIT microbiota, such as the age, feeding, and geography (Fig. 3a). For example, we observed significant clustering of the fecal samples into their respective groups in NMDS analysis (Fig. 5a–c), including the developmental stages (1, 6, and 12 months old; Table S3), feeding styles (indoor feeding and grazing; Table S4), and geo-locations (Yunnan, Sichuan, Guangxi, and Hainan provinces of China; Table S5); similar trends were found for both the bacterial/archaeal and viral genomes (Fig. 5a–c) and consistent with the multiple-factor PERMANOVA analysis (Fig. 3a).

We thus also explored the microbial taxa affected by these factors in more details. We identified a total of 350 bacterial/archaeal the genera shown in the figure are those that exhibited significantly abundant differences in at least one between-group comparisons, with 201, 99, and 114 genera that could be attributed to the age, feeding style, and geography, respectively (Fig. 5d). Interestingly, we observed significant overlaps among these groups of genera (54 genera showed significant relative abundance differences in all four factors). For example, feeding-associated genera were almost a subset of the age-associated ones, while they both overlapped significantly with the geography-associated ones (Fig. 5d). These results were in fact expected because all three factors were related to differences in the dietary structure. For example, goats older than 6 months would switch from milk-enriched to forage-enriched diet, causing significant increase of methane producers as well as cellulose-digesting species (Fig. 5e); the effects of which on the GIT microbiota would be like those between the grazing (grass-enriched diet) and indoor feeding (silage-enriched diet) types (Fig. 5e). We thus were tempted to speculate that the overlapped genera were related to the core traits of the goats such as the food digestion and nutrient absorption efficiencies and the methane emission, and would be universally important. Conversely, the factor-specific genera such as those associated only with the age would play important roles during goat development.

#### Host prediction of the GMMC virome and identification lytic viruses targeting methane producers

**Fig. 6:**
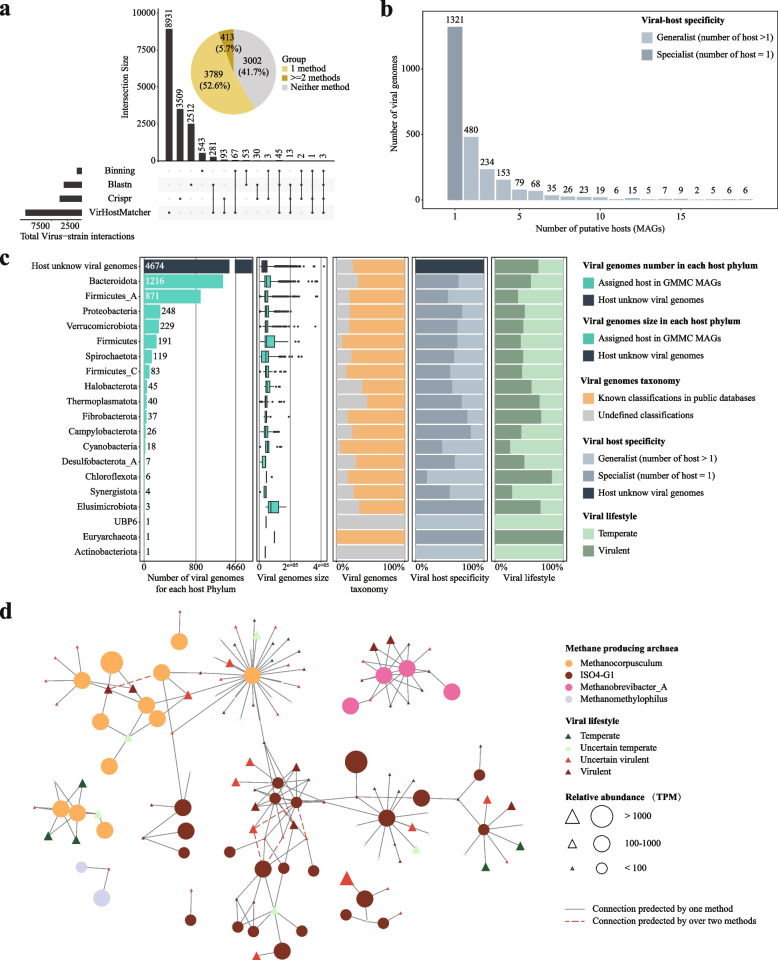
Host prediction of GMMC virome and identification of lytic viruses that target methane producers. **a** Overlaps of the prediction results on viral-host interactions using four different methods. The size is the number of viral-host interactions. Pie chart showing the proportion of viral genomes whose host(s) could predicted by these methods. **b** Distributions of the viruses as a function of their number of predicted hosts. The viruses could be divided into specialist (number of hosts = 1) and generalist (number of hosts > 1). **c** Distribution and characteristics of the viruses as a function of the taxonomic classification of their hosts, including the genome size, annotation rate, host specificity, and lifestyles. The lifestyles were predicted using DeePhage [76] and classified into two groups (virulent: score ≥ 50, temperate: score < 50). **d** Interaction network between viruses and methane producers (i.e., archaea). The solid line and the red dashed line indicate the connections predict by one method and by two methods, respectively

Because most viruses have host ranges at species levels [104], they are ideal tools for precision manipulation of goat GIT bacteria and archaea. We thus also predicted hosts for the GMMC viruses using four different methods, including CRISPR-spacer and homology-based methods, a VirHostMacher tool [105], and a binning-based method. In total, 4202 viral genomes (58.3% out of total) could be assigned to their bacterial/archaeal hosts (i.e., MAGs in the GMMC) by at least one method. We observed little overlaps among the methods in terms of viral-host relationships, consistent with previous results [106]. In total, only 5.7% viral-host relationships were supported by two or more methods (Fig. 6a). Overall, 1321 viruses (31% out of the 4202 with predicted hosts) were predicted to have only one host and could be classified as specialist (Fig. 6b), whereas the rest of viruses were associated with two or more hosts and were classified as generalists. Among all the MAGs, 1216 in *Bacteroidota* were predicted to be hosts for the GMMC viruses, followed by *Firmicutes_A* (*n* = 871), *Proteobacteria* (*n* = 248), and *Verrucomicrobiota* (*n* = 229). At the genus level, the most assigned hosts were *Prevotella* (*n* = 552), followed by *Alistipes* (*n* = 500), *RF16* (*n* = 480), *F082* (*n* = 334), and *Akkermansia* (*n* = 196). Many of the functionally important genera were targeted by the viruses including *Prevotella* (cellulose-digesting genus [107]), *Alistipes*, *Akkermansia* (host immune function [108, 109]), and *RF16* (feed digestion [110]), suggesting important regulatory roles in the goat GIT microbial structures and functions.

Viruses could be ideal agents to suppress the growth of methane-producing species [111]; however, so far only a few methanogen viruses have been identified (ref [112] and references therein). We thus screened all the viruses and identified a total of 104 that targeted the methanogens (Fig. 6d). Sixty-eight of the viruses were putative lytic ones (virulent or uncertain virulent) according to a DeePhage tool [113] (Methods) and could target the methanogens from all the four genera identified in this study. In addition, five viral-host relationships were supported by two or more methods, including those between four lytic viruses and three methanogens (Fig. 6d). There results added support for future efforts on targeted isolation of the viruses and experimental validation of their virulence against the methanogens.

## Discussion

Goats are important ruminant livestock whose microbiomes along the gastrointestinal tract (GIT) are known to play important roles for digestion, absorption, and beyond [1–3]. Despite recent significant advances in microbiome studies, a comprehensive survey on the goat microbiomes covering GIT sites, developmental stages, feeding styles, and geographical factors is still unavailable. In this study, we filled this gap by collecting and expensively analyzing a goat metagenomic dataset consisting of 497 samples, covering ten GIT sites, three developmental stages, two feeding styles, and four geographical locations. Based on this dataset, we built a goat multi-kingdom microbiota catalog (GMMC) consisting 4004 bacterial, 71 archaeal, and 7204 viral genomes, and annotated a total of 4,817,256 non-redundant protein-coding genes. The GMMC genomes contained significant proportions of novel ones, especially when compared with the two recent goat microbiome datasets [9, 24] (Fig. 1g) and significantly increased the coverage of the goat microbiome sequencing reads over the public datasets (Fig. 1e).

In addition to the data novelty, our analyses on the GMMC data also revealed several important implications that could be generalized to other ruminants.

First, we revealed a diet driven microbial community dynamic pattern along the goat GIT that was associated with goat intestine food digestion and absorption capacities and disease risks. More specifically, cluster 1 was mainly driven by silage feeding and associated with lower digestive burdens (e.g., processed feed requires less chew and intestinal capacities for digestion and absorption) of the large intestine but high enteritis risks for the jejunum (Fig. 3d, e, Fig. S6); conversely, cluster 2 was driven by the grass feeding and associated with higher digestive burdens of the large intestine. Future work is thus needed to determine if similar patterns could be observed in other ruminants.

Second, we showed that factors including age, feeding style, and geography also exerted significant impacts on the intestinal microbiota but most of the affected microbial taxa were directly or indirectly related to those affected by the feeding styles. For example, 54 out of the 350 differential genera related to at least one of the factors such as GIT site, age, and geography overlapped with those related to the feeding styles (Fig. 5d), especially those involved in methane production and cellulose digestion. We thus speculate that the overlapped genera were related to the core traits of the goats such as the food digestion and nutrient absorption efficiencies and the methane emission, and would be universally important, whereas the factor-specific genera such as those associated only with the age would play important roles during goat development. Given the importance of GIT microbiota in ruminants, we expect to find similar patterns in all these animals.

Last, we obtained 68 lytic viruses targeting methane-producing species in all four archaea genera by mining the GMMC catalog (Fig. 6d; Table S8). Previous studies suggested that viruses targeting methane producers could be useful to reduce methane emissions [111] but we lacked methods to identify such viruses at large scales (ref [112] and references therein). Our results thus provided a feasible method and would encourage researchers to mine similar resources for other important ruminants including buffalo [10] and cattle [22].

Despite the aforementioned advancements, it is important to acknowledge the limitations of our dataset, primarily attributed to the relatively short read length of the mNGS platform used. Recent studies have demonstrated significant improvements in assembly quality and the ability to obtain higher proportions of complete MAGs through long-read sequencing platforms such as PacBio and Nanopore, as observed in studies conducted on chickens [114] and humans [115, 116]. While our study achieved a considerable proportion of high-quality MAGs (47%), there is potential for further improvement by leveraging the capabilities offered by 3rd-generation sequencing platforms.

Together, our assembly and analyses of the GMMC catalog provided functional insights of the goat GIT microbiota that could potentially apply to other ruminants and pave the way to microbial interventions for better goat and eco-environmental qualities.

### Conclusions

We have provided the goat multi-kingdom microbiome catalog (GMMC) including bacterial, archaeal, viral genomes, and encoded-proteins and identified key microbial taxa important for key traits of the goat and their influencing factors, and many lytic viruses that could target methane producers.

### Supplementary Information

**Additional file 1: Supplement Table S1-S9. Table S1.** Detailed sampling information for 497 samples, **Table S2.** Number of gastrointestinal tract samples, **Table S3.** Number of age samples, **Table S4.** Number of feeding styles samples, **Table S5.** Number of different areas samples, **Table S6.** Statistics of assembly results of 497 samples, **Table S7.** Statistical information and classification level annotation of 4,075 MAGs, **Table S8.** Statistical information and classification level annotation of 7,204 phage contigs, **Table S9.** The CT value of different gastrointestinal tract samples.

**Additional file 2: Supplement figures S1-S12. Fig. S1.** Overview of the overall strategy and datasets employed for GMMC, **Fig. S2.** Viruses annotation proportion, **Fig. S3.** The species difference and classification between GMMC MAGs and published ruminant catalog, **Fig. S4.** The phylogenetic relationship among the viral genomes in the GMMC and their taxonomic classification, **Fig. S5.** Rarefaction analysis of the unique number of non-redundant proteins and viral genomes, **Fig. S6.** The state of intestinal contents of goats fed silage diet and grass, **Fig. S7.** The relative abundance of methane production and cellulose digestion genus in different GIT site, **Fig. S8.** The different genus in different GIT site which significantly positive correlation with GHs classification in goats were sorted according to the highest relative abundance GIT site, **Fig. S9.** The relative abundance of methane production and cellulose digestion genus in different age, feeding style and geography, **Fig. S10.** The standard curve of the real-time quantitative polymerase chain reaction (qPCR), **Fig. S11.** Purity (see Methods) of four methods in different taxonomy ranks, **Fig. S12.** Agreement (see Methods) between two methods in different taxonomy ranks.

## Data Availability

The raw sequencing data were submitted to the NCBI SRA database under the accession ID PRJNA723432; The goat multi-kingdom microbiome catalog (GMMC) catalog used in this study are available in the Figshare database under accession code 21,695,615 (https://doi.org/10.6084/m9.figshare.21695615.v1).
