# Supplementary material for: The multi-kingdom microbiome of the goat gastrointestinal tract
Source: Microbiome. 2023 Oct 2;11:219. doi: 10.1186/s40168-023-01651-6 (PMC10544373; doi:10.1186/s40168-023-01651-6)
Supplement: Supplementary file 3 — Additional file 2: Supplement figures S1-S12. Fig. S1. Overview of the overall strategy and datasets employed for GMMC, Fig. S2. Viruses annotation proportion, Fig. S3. The species difference and classification between GMMC MAGs and published ruminant catalog, Fig. S4. The phylogenetic relationship among the viral genomes in the GMMC and their taxonomic classification, Fig. S5. Rarefaction analysis of the unique number of non-redundant proteins and viral genomes, Fig. S6. The state of intestinal contents of goats fed silage diet and grass, Fig. S7. The relative abundance of methane production and cellulose digestion genus in different GIT site, Fig. S8. The different genus in different GIT site which significantly positive correlation with GHs classification in goats were sorted according to the highest relative abundance GIT site, Fig. S9. The relative abundance of methane production and cellulose digestion genus in different age, feeding style and geography, Fig. S10. The standard curve of the real-time quantitative polymerase chain reaction (qPCR), Fig. S11. Purity (see Methods) of four methods in different taxonomy ranks, Fig. S12. Agreement (see Methods) between two methods in different taxonomy ranks. [file 40168_2023_1651_MOESM2_ESM.pdf]

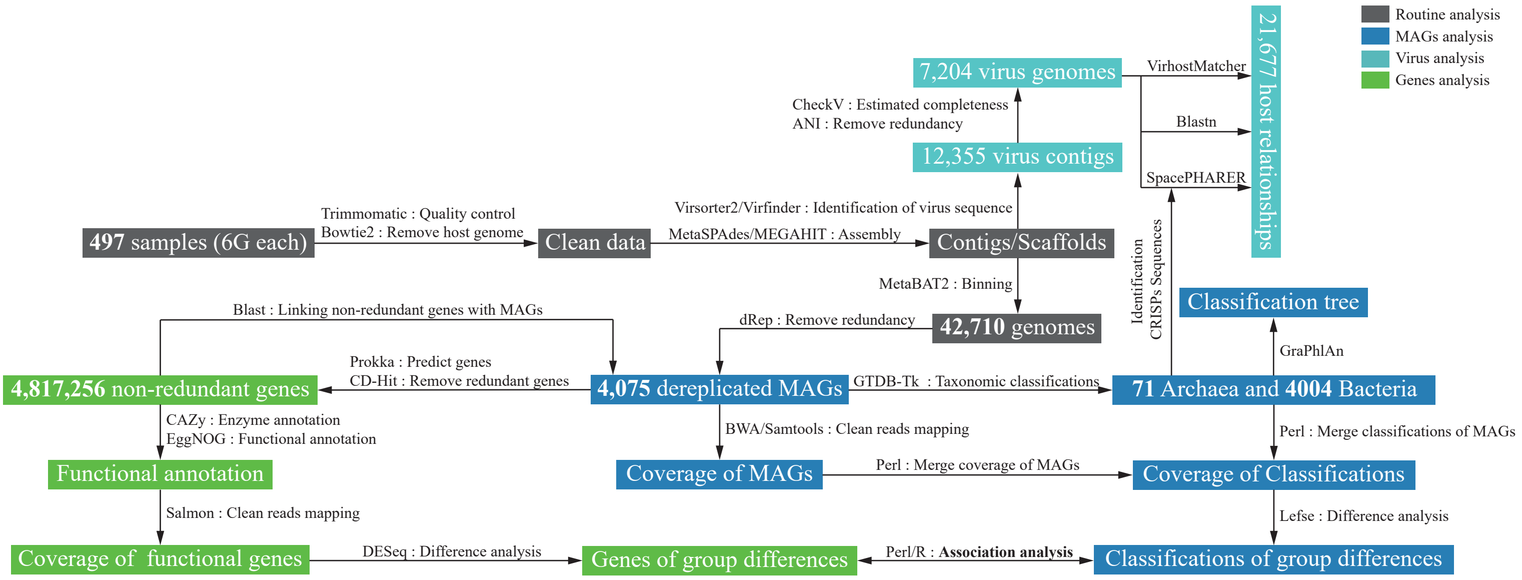

Figure S1. Overview of the overall strategy and datasets employed for GMMC.

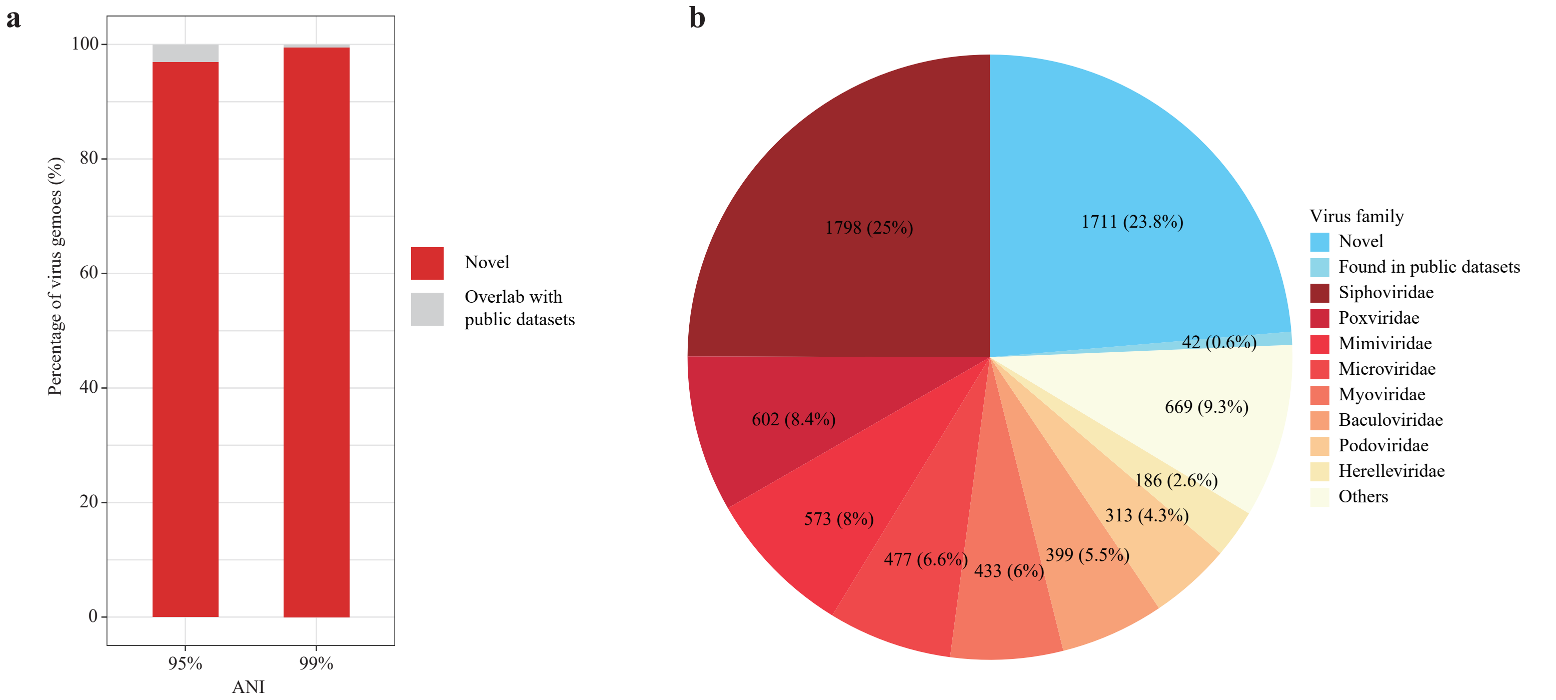

Figure S2. a Percentages of viral genomes in GMMC as compared with public datasets a 95% and 99% average nucleotide identity (ANI) thresholds (see Methods). **b** Pie chat showing the number of annotated family levels of the viruses and the number of viruses that could be found in public datasets (see Methods).

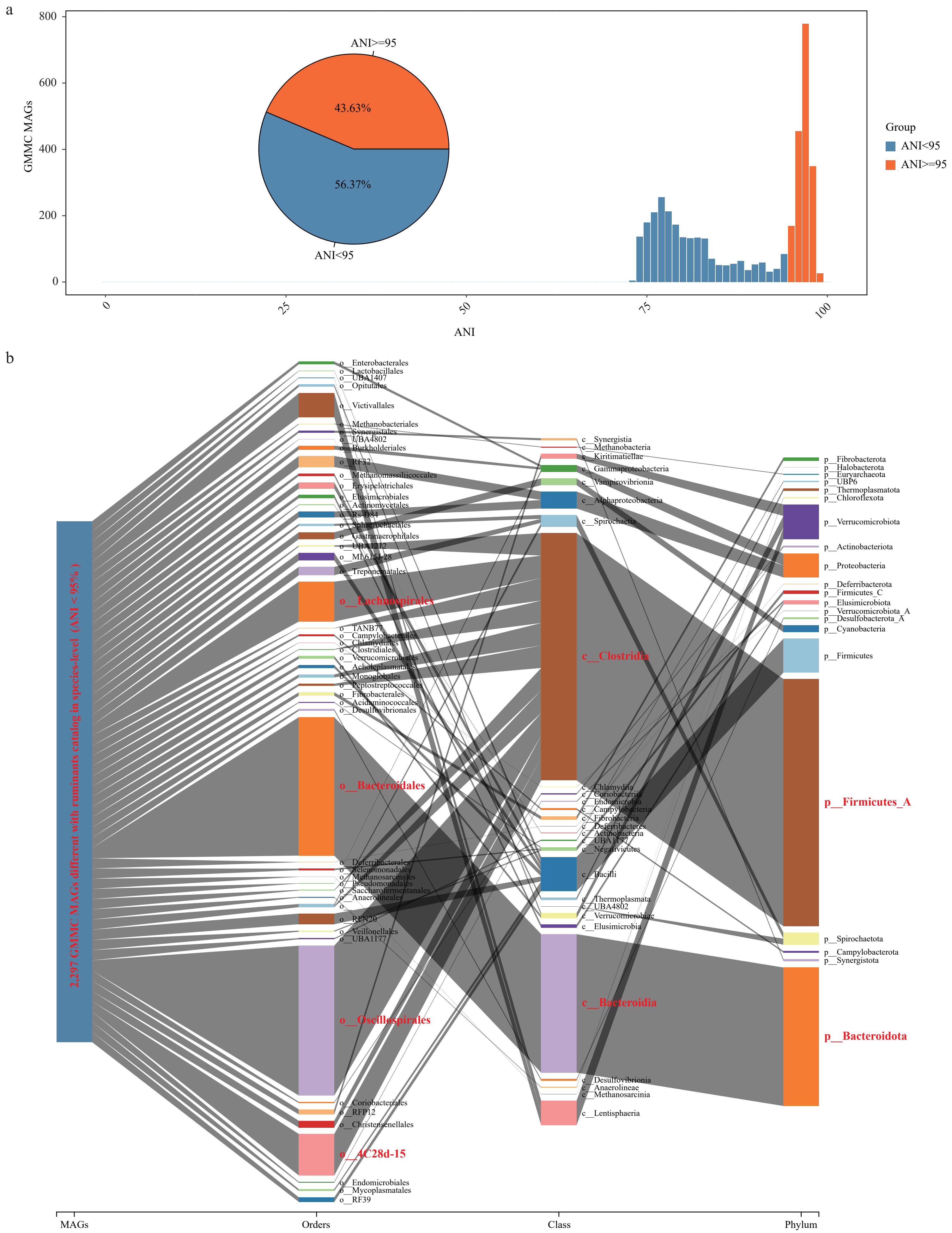

Figure S3. The species difference between GMMC MAGs and published ruminant catalog by Average Nucleotide Identity (ANI). a Distribution of ANI in GMMC MAGs compared with ruminant catalog. b The classification (orders, class and phylum) of 2,297 GMMC MAGs different with ruminants catalog in species-level.

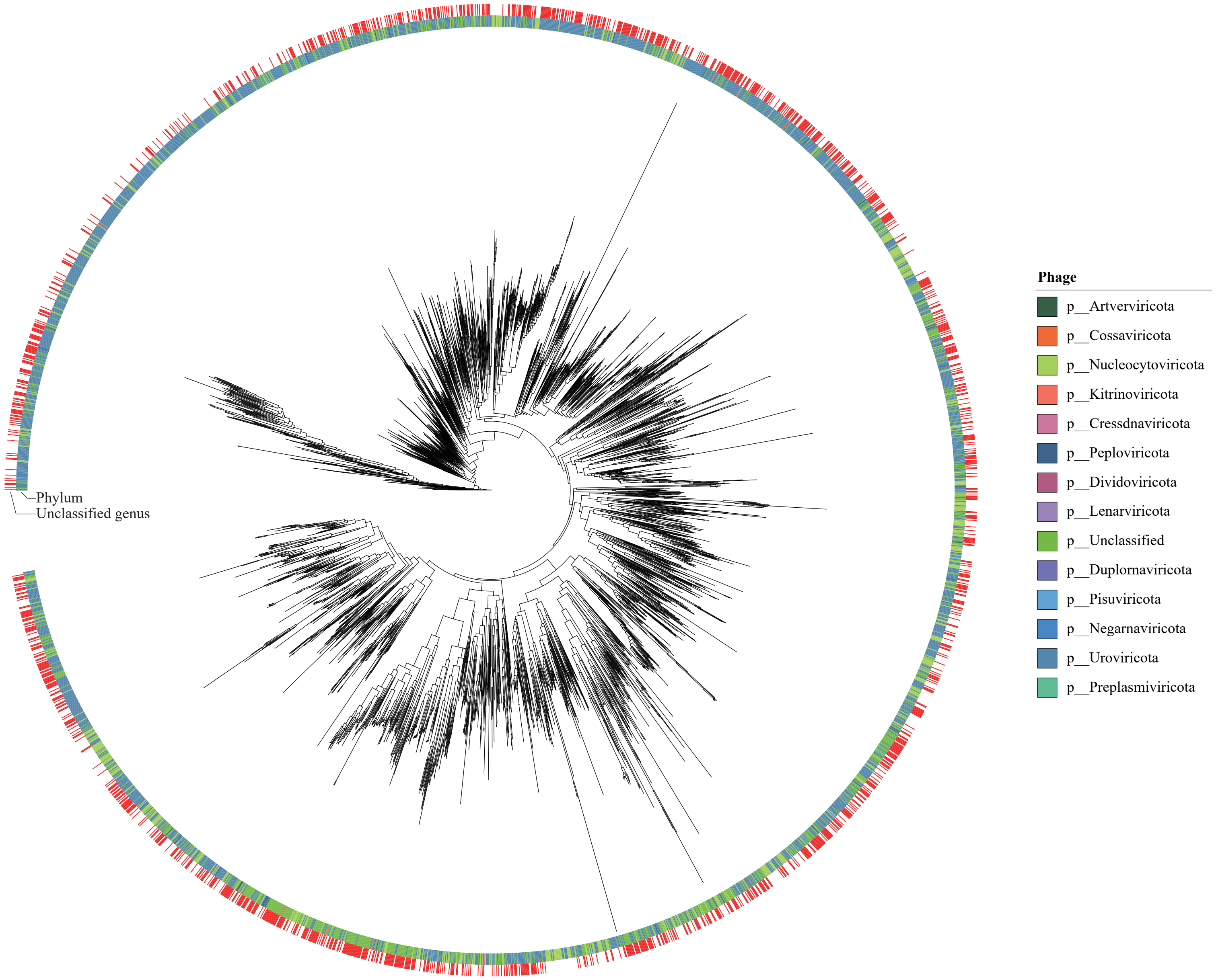

Figure S4. The phylogenetic relationship among the viral genomes in the GMMC and their taxonomic classification. The annotations from inside to outside represent annotations of species level (different colors represent different phyla), unclassified genus (in red).

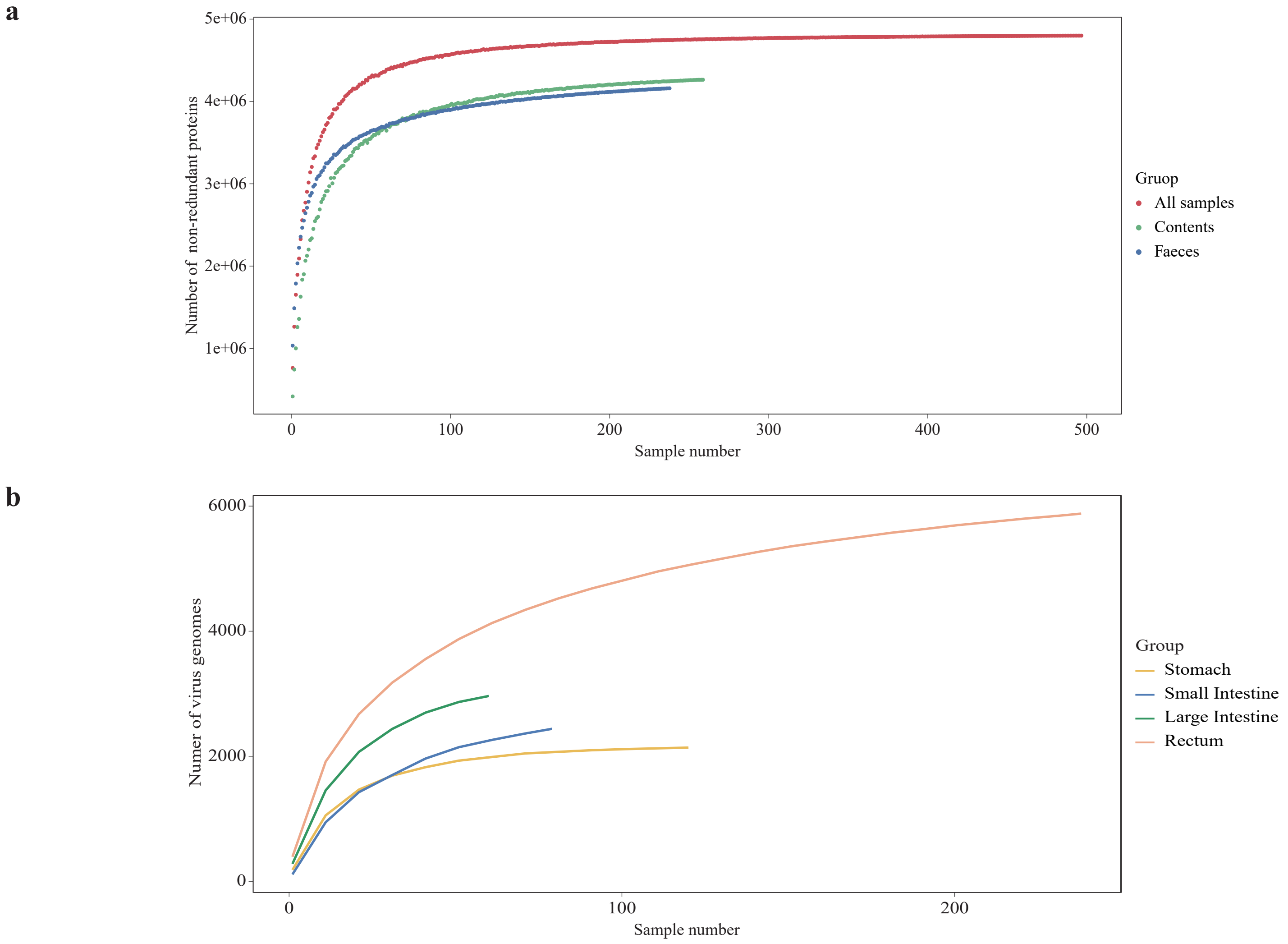

Figure S5. **a** Rarefaction curves of detected non-redundant proteins. The rarefaction curve is calculated using all samples, fecal samples and content samples respectively. **b** Rarefaction analysis of the unique number of virus genomes (Y-axis) as the function of samples (X-axis) in different intestinal locations of goats.

a

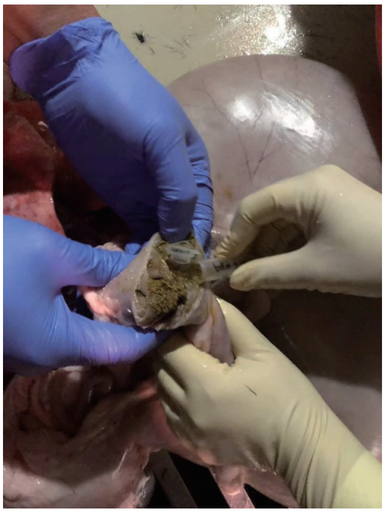

a

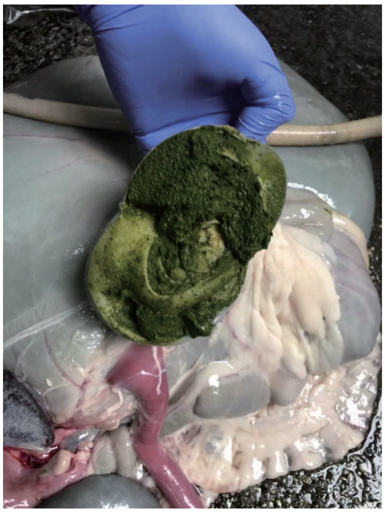

Figure S6. **a** The state of intestinal contents of goats fed silage diet. **b** The intestinal contents of goats fed with grass.

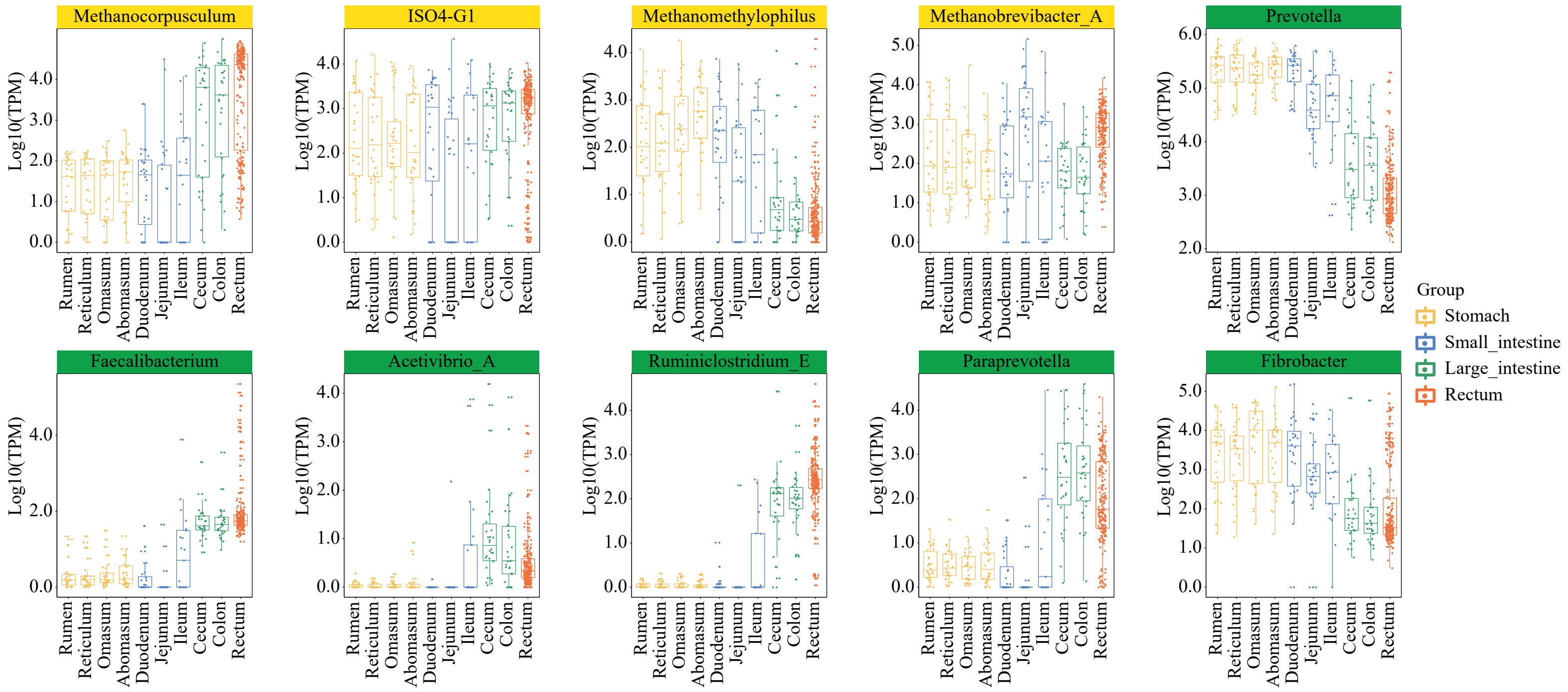

Figure S7. The relative abundance (using the TPM value after log10 conversion) of methane production and cellulose digestion genus in different GIT sites, the relative abundance ranges from 0 to 1. Yellow and green represent methane production and cellulose digestion functional genus respectively. Differential taxa were identified between two groups using LEfSe (see Materials and Methods); here, Wilcoxon Rank Sum test were used to show the statistical significance between groups. ns: no significance, * P < 0.05, ** P < 0.01, *** P < 0.0001, **** P <0.0001.

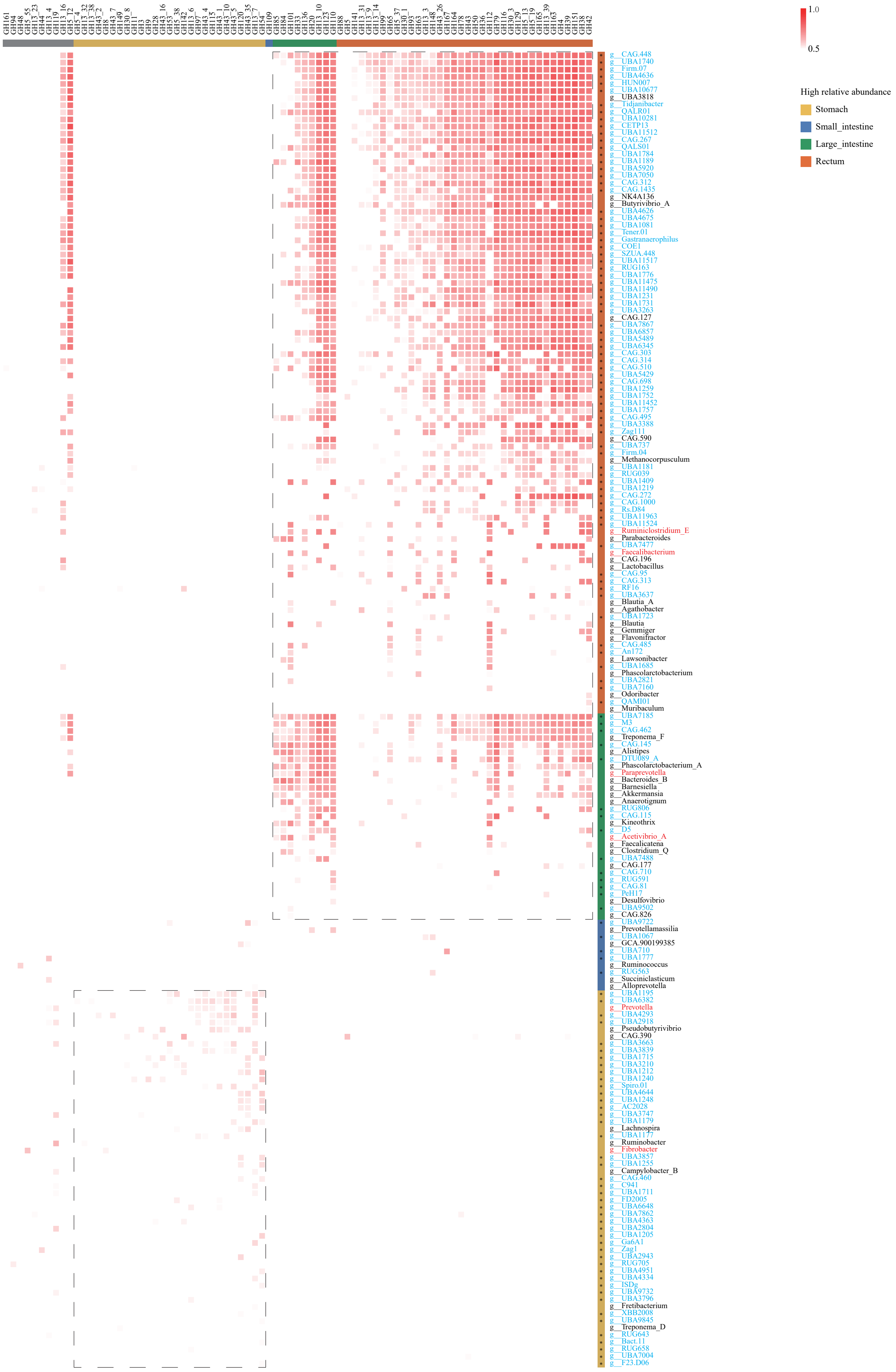

Figure S8. The different genus in different GIT sites which significantly positive correlation with GHs classification in goats were sorted according to the highest relative abundance GIT site. The color of heatmap indicates the correlation coefficient between genus and GHs classification (R >= 0.5), only significant positive correlation results are retained (P <=0.05, R >=0.5). Pie chart shows the location of the GIT sites with the highest abundance of genus and GHs classification. * represents the genus without functional background at present.

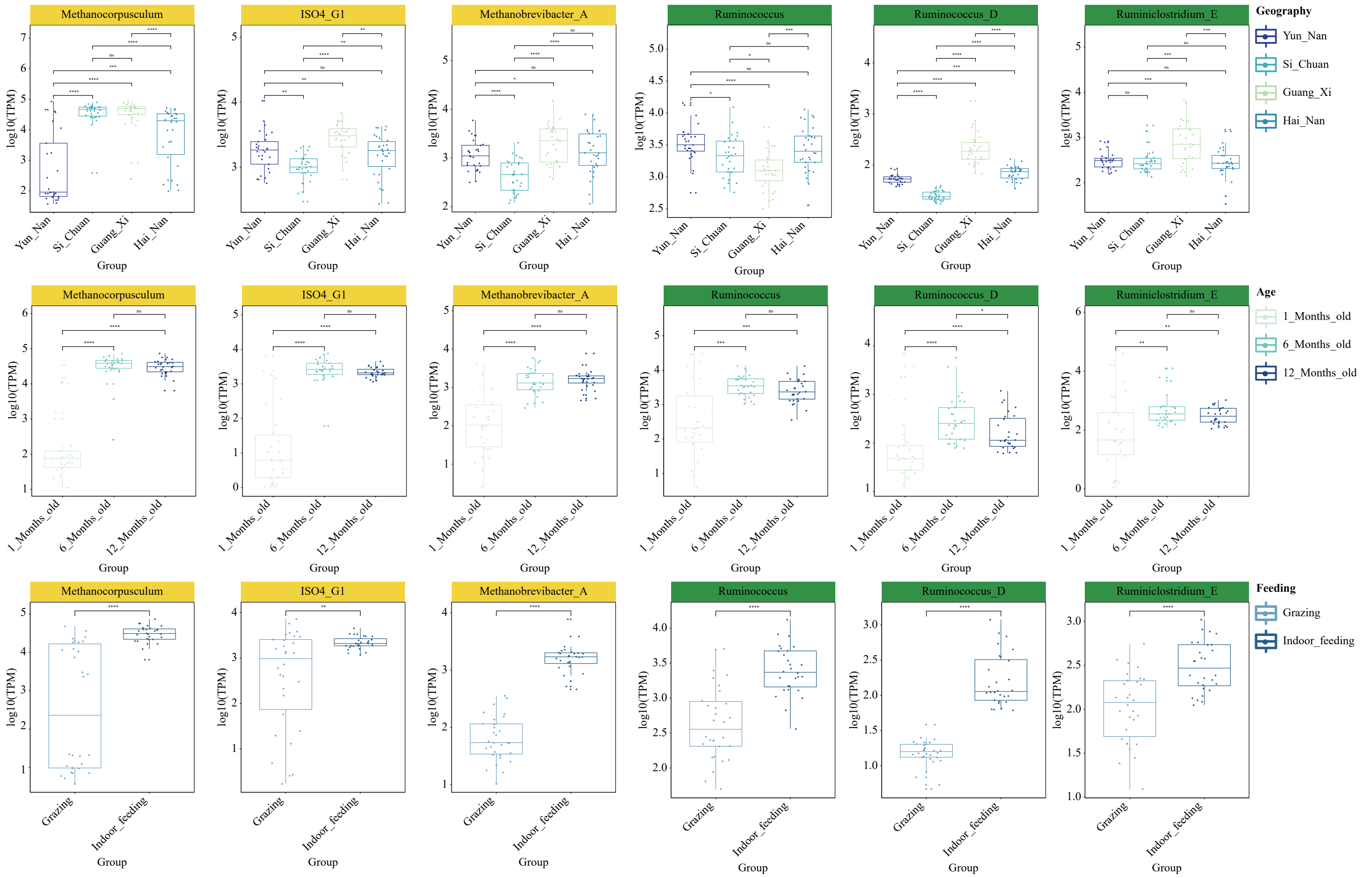

Figure S9. The relative abundance (using the TPM value after log10 conversion) of methane production and cellulose digestion genus in different age, feeding style and geography, the relative abundance ranges from 0 to 1. Yellow and green represent methane production and cellulose digestion functional genus respectively. Differential taxa were identified between two groups using LEfSe (see Materials and Methods); here, Wilcoxon Rank Sum test were used to show the statistical significance between groups. ns: no significance.
* P < 0.05, ** P < 0.01, *** P < 0.0001, **** P <0.0001.

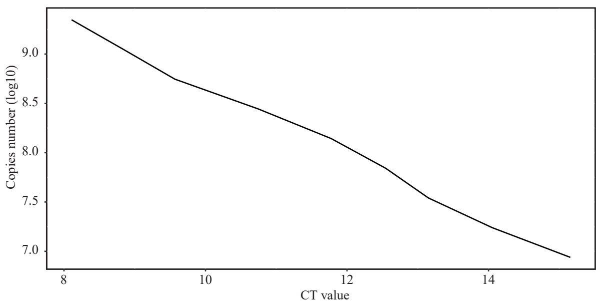

Figure S10. The standard curve of the real-time quantitative polymerase chain reaction (qPCR).

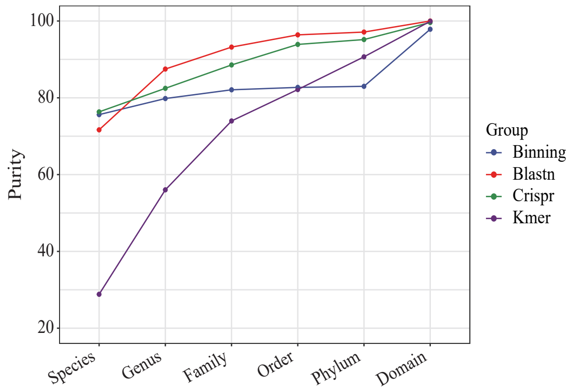

Figure S11. Purity (see Methods) of four methods in different taxonomy ranks.

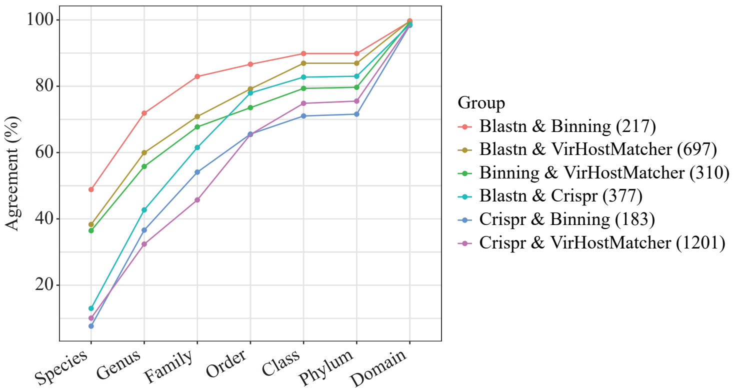

Figure S12. Agreement (see Methods) between two methods in different taxonomy ranks. The numbers in parentheses indicate the number of viruses predicted to the host by both methods.
